# The effect of nitrogen fertilization and the use of biostimulators on the chemical composition of grains of two varieties of corn (*Zea mays* L.)

**DOI:** 10.1371/journal.pone.0311560

**Published:** 2024-12-30

**Authors:** Marek Niewęgłowski, Anna Sikorska, Marek Gugała, Ewa Krasnodębska, Krystyna Zarzecka

**Affiliations:** 1 Faculty of Agricultural Sciences, University of Siedlce, Siedlce, Poland; 2 Department of Agriculture, Ignacy Mościcki University of Applied Sciences in Ciechanów, Ciechanów, Poland; 3 Family Farm in Niemirki 54, Jabłonna Lacka, Poland; Universidad Autónoma Agraria Antonio Narro, MEXICO

## Abstract

The aim of the research was to determine the impact of the use of biostimulators and different nitrogen doses on the yield quality of two varieties of corn grown for grain. The field experiment was carried out in 2015–2017 on an individual farm located in north-eastern Poland (52°30’N and 22°26’E). The following factors were examined in the experiment: group I—two corn varieties: PR38N86 (280 FAO); P8400 (240 FAO) group II—four doses of nitrogen fertilization: control treatment—without nitrogen application (0 kg·ha^-1^ N) nitrogen doses—80 kg·ha^-1^ N, 120 kg·ha^-1^ N, 160 kg·ha^-1^ N, group III—four types of biostimulators used: (1) control treatment–without the use of a biostimulator, (2) biostimulator containing sodium ortho-nitrophenol, sodium para-nitrophenol, 5-nitroguaiacol sodium, (3) biostimulator containing potassium para-nitrophenolate, potassium ortho-nitrophenolate, potassium 5-nitrovacollate, (4) biostimulator containing molybdenum, zinc. Different doses of nitrogen used in the experiment contributed to an increase in the total protein content in the grain of the tested corn varieties compared to the control treatment. The highest significant increase in protein content by an average of 18.96 g∙kg^-1^ was obtained after the application of 160 kg N∙ha^-1^, and after the application of 120 kg N∙ha^-1^ the value of this feature increased on average by 17.18 kg N∙ha^-1^. Nitrogen fertilization reduced the starch content and increased the fat content in the tested corn varieties. The lowest starch content was found after the application of 120 kg N∙ha^-1^ and 160 kg N∙ha^-1^, while the highest fat content was found in grain tested with 120 kg N∙ha^-1^. The highest protein content was demonstrated after the use of a biostimulator containing sodium ortho-nitrophenol, sodium para-nitrophenol, and sodium 5-nitroguaiacol. The biostimulators used increased the starch and fat content in the grain of the tested varieties, and the differences between them were statistically insignificant. After using a biostimulator containing sodium ortho-nitrophenol, sodium para-nitrophenol, sodium 5-nitroguaiacol, the protein content increased by an average of 6.0%. The biostimulators used increased the starch and fat content in the grain of the tested varieties, and the differences between them were statistically insignificant. The medium-early variety with FAO 240 was characterized by a higher content of total protein (on average by 7.95 g∙kg^-1^) and crude fat (on average by 1.5 g∙kg^-1^) compared to the medium-late variety. All tested varieties were distinguished by the highest concentration of total protein and crude fat in the second, optimal year of the study. The lowest protein content was found in the last year of the study with the highest annual rainfall and the lowest average air temperature, and crude fat content was found in a relatively dry growing season. The opposite tendency was obtained in the case of starch content in the grain of the tested varieties.

## Introduction

Cultivating corn for grain in Poland, despite significant achievements in breeding and some climate warming, is recommended in regions with more favourable conditions for this type of use [1]. Among many factors (weather conditions, selection of an appropriate variety with an appropriate FAO number) limiting the yield of corn grain [2, 3], the basic element of agrotechnics, depending on habitat conditions, is nitrogen fertilization. The amount of corn grain yield depends on the availability of easily digestible nutrients, for this reason, mineral fertilization is most often used, especially with nitrogen [4–7]. The use of large doses of nitrogen, especially on light soils, poses a threat to the environment. In turn, reducing the level of fertilization with this element prevents the use of the production potential of currently cultivated corn varieties and leads to reduced yields [4]. According to the research by Kruczek [8] and Jankowiak et al. [9], optimal nitrogen doses for corn grain yield range from 90 to 150 kg·ha^-1^ N. Nitrogen fertilization determines the content of nutrients in grain. According to Księżak and Bojarszczuk [10], the increasing doses of nitrogen cause an increase in the protein content both in the vegetative mass of corn and in the grain. Bogucka et al. [11] in their research proved that the highest protein yield in grain yield (1.02 Mg·ha^-1^) can be obtained with fertilization of 240 kg·ha^-1^ N.

In Poland, corn is cultivated on a large scale, but the yields obtained are not always satisfactory due to unfavourable climatic and soil conditions, especially those occurring in the initial phases of corn growth and development [12]. At average air temperatures ranging from 5 to 12°C, the activity of young corn roots to absorb ions, especially phosphorus and total nitrogen ions, is weakened [13]. Therefore, it is justified to use biostimulators in the early stages of development, and even to treat grain with biostimulators [14].

According to Michalski et al. [15] and Matysiak et al. [16], biostimulators increase the tolerance of plants to harmful environmental stimuli, especially drought, hence they are increasingly used in agricultural practice. Similarly, research by Pusz and Pląskowska [17] and Kozak [18] confirm that biostimulators are an effective way to prevent or reduce the adverse impact of environmental stresses on the amount and quality of crops. Their basic task is to participate in the regulation of life processes at the level of a cell, organ or the entire organism [19]. These are products of natural origin (plant or animal) safe for human health and the environment. Biostimulators contain active substances such as plant hormones, enzymes, macro- and microelements, as well as other substances that stimulate plant growth and development [20]. Biostimulators improve the uptake of minerals and stimulate the development of roots and leaves. Few studies prove that they can also reduce the occurrence of pests [21] and reduce the infection of plants by pathogens [22] and consequently contribute to improving the productivity of crops in the quantitative and qualitative sense [16].

The aim of the research was to determine the impact of the use of biostimulators and different nitrogen doses on the yield quality of two varieties of corn grown for grain. It should be emphasized that inappropriate and excessive use of chemical fertilizers has a negative impact on the natural environment, contributing to soil and groundwater contamination, reducing soil fertility, causing leaching of macroelements and increasing the availability of toxic forms of aluminium and manganese. Hence, intensive research is being carried out on the use of alternative means to reduce the doses of mineral fertilizers.

The research was carried out to verify the hypothesis that an appropriate dose of nitrogen and eliminating unfavourable weather conditions during plant growth through the use of growth biostimulators will have a positive effect on the quality of corn grain. The conducted research will allow for the recommendation of optimal fertilization in variable climatic conditions in the research area.

## Material and research methods

### Field experiment

The field experiment was carried out in 2015–2017 on an individual farm located in north-eastern Poland, village Niemirki (52°30’N and 22°26’E**)**. The experiment was set up in a split—split—plot arrangement with three repetitions. The area of one harvest plot was 30 m^-2^. The following factors were examined in the experiment:

group I–two corn varieties:

1. PR38N86 (mid-late hybrid variety 280 FAO)
2. P8400 (mid-early hybrid variety 240 FAO)

group II–four doses of nitrogen fertilization:

1. control treatment–without nitrogen application (0 kg N·ha^-1^)
2. nitrogen dose– 80 kg·ha^-1^ N (applied once before sowing)
3. nitrogen dose– 120 kg·ha^-1^ N (applied once before sowing)
4. nitrogen dose– 160 kg·ha^-1^ N (applied once before sowing)

group III–four types of biostimulators used:

1. control treatment–without the use of a biostimulator,
2. biostimulator containing sodium ortho-nitrophenol, sodium para-nitrophenol, 5-nitroguaiacol sodium: I term– 4^th^ phase of the leaf (Biologische Bundesanstalt, Bundessortenamt und Chemische Industrie—BBCH 14), II term– 8^th^ phase of the leaf (BBCH 18) at doses of 0.60 dm^3^∙ha^-1^,
3. biostimulator containing potassium para-nitrophenolate, potassium ortho-nitrophenolate, potassium 5-nitrovacollate: I term– 4^th^ phase of the leaf (BBCH 14) at a dose of 1.00 dm^3^∙ha^-1^, II term– 8^th^ phase of the leaf (BBCH 18) at a dose of 0.60 dm^3^∙ha^-1^,
4. biostimulator containing molybdenum, zinc: I term– 6^th^ phase of the leaf (BBCH 16) at a dose of 2.00 dm^3^∙ha^-1^.

The experiment was carried out on soil classified according to the IUSS Working Group WRB [23], to the Haplic Luvisol group, sandy, belonging to the very good rye soil complex, valuation class Iva. The soil was characterized by a high content of available forms of potassium, phosphorus and magnesium and an average content of mineral nitrogen in the 0–30 cm soil layer and in the 30–60 cm soil layer. The soil pH in the years of study was neutral and ranged from June 7 to December (Table 1). The analysis of the chemical properties of the soil was carried out in the laboratory of the District Chemical and Agricultural Station in Rzeszów.

**Table 1:** The nutrient content in the soil in 2015–2017.

Soil chemical composition	Unit	Years of research		
		2015	2016	2017
Mineral N content 0–30 cm	kg ∙ ha^-1^	65.24	64.80	64.91
Mineral N content 30–60 cm	kg ∙ ha^-1^	63.35	64.50	63.97
Average content of P_2_O_5_	mg∙100 g^-1^ of soil	21.05	21.35	20.15
Average content of K_2_O	mg∙100 g^-1^ of soil	26.00	26.75	25.95
Average content of Mg	mg∙100 g^-1^ of soil	6.95	7.05	7.90
Soil pH	1 mol∙dm^-3^ KCl	7.12	7.07	7.06

Grain corn was the forecrop for corn in particular years. After harvesting the forecrop, in order to crush the crop residues and mix them with the soil, a set of post-harvest cultivations was made using a plate dish, and then winter ploughing was used at a depth of 25.0 cm. In early spring, a tine harrow was used to even out and stop evaporation and accelerate soil heating. Then, potassium fertilization was applied in the form of potassium salt at a dose of 250 kg∙ha^-1^, i.e. 124.5 kg∙ha^-1^ K. Before sowing corn, nitrogen fertilization in the form of urea was applied. Corn was sown with a pneumatic precision seeder with row fertilization (triple superphosphate 46% - 71.8 kg∙ha^-1^ i.e. 33 kg∙ha^-1^ P) in rows with a spacing of 75 cm, maintaining a planting density of 8 plants∙m^2^.

Corn was sown at the optimal date recommended for this region (in 2015 –April 23, 2016 –April 23, 2017 –May 8 (the delay was due to unfavourable meteorological conditions).

Chemical protection against weeds and pests was applied in accordance with the recommendations of good agricultural practice.

The certified seed was obtained from Pioneer. The study was in accordance with relevant institutional, national, and international guidelines and legislation.

Corn was harvested when the grain was fully ripe in the first decade of October. The cobs collected from 1m^-2^ were left for about 3 weeks to dry. The grains from the cobs from each row were poured together to form a collective sample.

### Meteorological conditions

There were various weather conditions during the years of research (Table 2). Based on the calculated Selyaninov hydrothermal coefficient, the first growing season according to Skowera [24] was characterized by optimal atmospheric conditions (K = 1.41). In the first year of the study, the average air temperature was 0.1°C lower than the long-term average, and the sum of precipitation was 332.1 mm and was 1.0% lower than the long-term average. The next growing season was characterized by dry conditions during the development and growth of corn (K = 1.12). In April, conditions were quite dry, in May–optimal, and in October–extremely humid, while in June–dry, in July and August–very dry, and in September–extremely dry. In the second year of the study, the sum of precipitation was 64.7 mm lower compared to the sum of precipitation from the long-term period, and the aid temperature was 0.5°C higher than the long-term average. The last growing season was characterized by moderately wet weather conditions (K = 1.41), however, there were months with extreme conditions from very dry in July to quite dry in June and August. In the third year of the study, the average air temperature was 0.2°C lower than the long-term average, and the sum of precipitation was 378.4 mm and was 43 mm higher than the long-term average.

**Table 2:** Characteristics of climatic conditions in 2015–2017 (Zawady Agricultural Experimental Station, Poland).

**Growing season**	**Months**									
	**IV**		**V**		**VI**	**VII**	**VIII**	**IX**	**X**	**IV-X**
**Air temperature (** ^**o**^ **C)**										Mean
2015	8.2	12.3			16.5	18.7	21.0	14.5	6.5	**13.9**
2016	9.1	15.1			18.4	19.1	18.0	14.9	7.0	**14.5**
2017	6.9	13.9			17.8	16.9	18.4	13.9	9.0	**13.8**
Multiyear mean (1996–2010)	**8.0**	**13.5**			**17.0**	**19.7**	**18.5**	**13.5**	**7.0**	**14.0**
**Precipitation (mm)**										**Sum**
2015	30.0	100.2			43.3	62.6	11.9	47.1	37.0	**332.1**
2016	28.7	54.8			36.9	35.2	31.7	13.6	69.8	**270.7**
2017	59.6	49.5			57.9	23.6	54.7	80.1	53.0	**378.4**
Multiyear total (1996–2010)	**33.6**	**58.3**			**59.6**	**57.5**	**59.9**	**42.3**	**24.2**	**335.4**
**Sielianinov hydrothermal coefficient** *										**Mean**
2015	1.35	2.91		0.84		1.21	0.20	1.20	2.15	**1.41**
2016	1.08	1.47		0.72		0.64	0.62	0.28	3.02	**1.12**
2017	3.82	1.52		1.07		0.47	1.01	1.92	2.36	**1.74**

* Coefficient value [24]: Extremely dry (ss) k≤0.4; Very dry (bs) 0.4–0.7; Dry (s) 0.7–1.0; Rather dry (ds) 1.0<k≤1.3; Optimal (o) 1.3<k≤1.6; Rather wet (dw) 1.6<k≤2.0; Wet (w) 2.0<k≤2.5; Very wet (bw) 2.5<k≤3.0; Extremely wet (sw) k>3.0

### Chemical analyses

The crude fat content (g∙kg^-1^) was determined by the Soxhlet method, which involves extracting fat with petroleum ether in a Soxhlet apparatus and determining its amount by weight, according to PN-76/R-64753.

The total protein content (g∙kg^-1^) was determined by the Kjeldahl method, which involves converting protein nitrogen into ammonium sulphate (VI) using concentrated sulphuric acid in the presence of a catalyst, locating the solution, distillation and titration with hydrochloric acid–ammonia bound with boric acid, according to PN-EN ISO 5983–2.

Starch content (g∙kg^-1^) was determined on an Infratec^TM^ 1241 whole gran analyser.

Chemical analyses of grains were performed in the chemical-technological laboratory of the Experimental Variety Assessment Station in Słupia Wielka.

### Statistical analysis

The research results were analysed statistically using the variance analysis. The significance of the sources of variability was tested using the Fischer-Snedecor “F” test, and the significance of differences was assessed at the significance level of p = 0.05 between the compared means using Tukey intervals. Statistical calculations were made based on a proprietary algorithm written in Excel in accordance with a mathematical model.

## Research results and discussion

### Protein content in corn grain

The statistical analysis of the test results showed a significant impact of **varieties** on the protein content in the grain of the tested corn varieties (Table 3), which is consistent with the research results of other authors [25, 26]. The relationship between the protein content in the grain of individual varieties is also confirmed by the research of Korniewicz et al. [27]. These authors showed that the grain of early hybrids contained an average of 10.45% of the dry mass of protein, and medium-early hybrids on average 10.28% of the dry mass. It is consistent with the results of own research, which found that the value of this feature in the medium-late variety PR38N86 was lower on average by 7.95 g∙kg^-1^ compared to the variety with a lower FAO number–P8400. In turn, Bogucka et al. [11] did not prove significant differences between the tested corn varieties, i.e. early Junak– 89.9 g·kg^-1^ d.m. and medium-early Boruta– 90.1 g·kg^-1^ d.m. However, in the study by Podkówka and Podkówka [28], greater of the value of the discussed feature was demonstrated in medium-late varieties compared to medium-early varieties, but these differences were statistically insignificant.

**Table 3:** Protein content in corn grain (g∙kg^-1^) depending on research factors.

Factors studied	Growing season			Average
	2015	2016	2017	
**VARIETIES **				
medium-late—PR38N86	82,19 ^b^	89,62 ^d^	68,16 ^e^	79,99 ^B^
medium-early—P8400	92,35 ^a^	103,55 ^c^	67,92 ^e^	87,94 ^A^
**NITROGEN DOSES**				
Control treatment	68.01 ^c^	88.31 ^d^	62.15 ^d^	**72.82** ^**D**^
80 kg·ha^-1^ N	85.47 ^b^	90.45 ^c^	67.85 ^c^	**81.26** ^**C**^
120 kg·ha^-1^ N	97.92 ^a^	101.98 ^b^	70.11 ^b^	**90.00** ^**B**^
160 kg·ha^-1^ N	97.68 ^a^	105.60 ^a^	72.07 ^a^	**91.78** ^**A**^
**TYPES OF BIOSTIMULATORS USED**				
Control treatment	82.14 ^d^	94.03 ^b^	66.48 ^b^	**80.88** ^**C**^
Biostimulator containing: sodium ortho-nitrophenol, sodium para-nitrophenol, 5-nitroguaiacol sodium	91.24 ^a^	97.94 ^a^	69.02 ^a^	**86.06** ^**A**^
Biostimulator containing: potassium para-nitrophenolate, potassium ortho-nitrophenolate, potassium 5-nitrovacollate	86.78 ^c^	97.50 ^a^	68.55 ^a^	**84.28** ^**B**^
Biostimulator containing: molybdenum, zinc	88.94 ^b^	96.87 ^a^	68.12 ^a^	**84.64** ^**B**^
** Means for years**	**87.27** ^**B**^	**96.58** ^**A**^	**68.04** ^**C**^	-

* The means for growing seasons, varieties, nitrogen doses, and types of biostimulators used, marked with capital letters A, B, C, differ significantly at p≤0.05. Means for interactions: years and varieties, years and nitrogen doses; years and types of biostimulators used, marked with lowercase letters a, b, c, d differ significantly at p≤0.05.

Księżak et al. [4] found that increasing the **nitrogen dose** from 0 to 200 kg·ha^-1^ N resulted in an increase in the protein content both in the vegetative mass of corn and in the grain. This is consistent with the results of own research, in which the highest value of this feature was obtained using 160 kg·ha^-1^ N, but it should be noted that this value was higher on average by 1.78 g kg^-1^ d.m. compared to the treatment where 120 kg·ha^-1^ N was used. Similar results were obtained by Bogucka et al. [11], where the highest protein yield in grain yield (1.02 Mg·ha^-1^) was obtained in corn fertilized with 240 kg·ha^-1^ N, but these differences were statistically insignificant. Similarly, Almodares et al. [29] obtained the highest value of this feature after applying 200 kg·ha^-1^, while Szulc [30] obtained significant differences in the total protein content depending on different nitrogen doses, with the highest protein content obtained when fertilizing with 120 kg·ha^-1^ N. Also in the studies of Kruczek [8] and Filipek-Mazur et al. [31], a significant effect of nitrogen dose on the discussed feature was found.

Own research showed that the **biostimulators** used in the experiment significantly increased the content of total protein in corn grains. The greatest significant increase in the value of this feature was demonstrated after the application of the preparation containing the active substance: sodium ortho-nitrophenolate, sodium para-nitrophenol and sodium 5-nitroguaiacol. Similar results were obtained by Abdel-Lattif et al. [32]. Different results were obtained by Kierzek et al. [33], who showed no significant effect of biostimulators containing amino acids in different application systems (twice together with the herbicide and separately), as well as the Asahi SL biostimulator on the protein content in corn grain. Also in the study by Michalski [34], the Biochikol biostimulator did not significantly affect the value of this feature.

The content of total protein in corn grain varied between the years of the study (Table 3), the highest value of the discussed feature–an average of 96.58 g∙kg^-1^ was obtained in the season which was characterized by higher sums of temperatures during the growing season than the average temperature over the long term and according to the Selyaninov coefficient it was quite dry. Similar research results were obtained by Szmigiel and Kiełbasa [26], who obtained the highest total protein content in corn grain harvested during the growing season characterized by the highest air temperature and moderate rainfall. However, Bogucka et al. [11] obtained the highest protein content in a very wet and warm year.

The research showed interaction between the years and the varieties tested. The grain of the medium-late and medium-early varieties had the highest protein content in a rather dry growing season (2016). In the last year of the study, when significant rainfall occurred and average air temperatures were higher than the long-term average, the varieties were characterized by the lowest value of this feature, and the differences between them were statistically insignificant.

Statistical calculations confirmed the differentia effect of nitrogen doses on protein content in changing climatic conditions during the research. In all years of the study, there was a significant increase in the protein content in corn grain under the influence of an increased dose of nitrogen compared to the control object. In the first growing season, the highest protein content was obtained under the influence of 120 kg∙ha^-1^N compared to the others, and the differences in the value of this feature after the application of 120 and 160 kg∙ha^-1^ N were insignificant. In the last two years of research, the highest value of the discussed feature was obtained as a result of using 160 kg∙ha^-1^ N.

The effect of biostimulators varied over the years of research. In all seasons, there was a significant increase in the protein content in corn grain under the influence of the bioregulators used compared to the control object. The highest protein content was found under the influence of a biostimulator containing sodium ortho-nitrophenol, sodium para-nitrophenol, and sodium 5-nitroguaiacol. In the second and third years of the study, no significant differences in protein content between biostimulators were found, except for the first, quite dry year of the study.

### Starch content in corn grain

Own research shows that the tested varieties differed significantly in starch content (Table 4). The highest value of the discussed feature was obtained in the medium-late variety PR38N86, which had an average of 2.18 g∙kg^-1^ higher starch content than the medium-early variety. Different results were obtained by Korniewicz et al. [27], who showed a higher starch content in hybrids with FAO number 190–210 (early) compared to FAO 250–270 (medium-late). A similar relationship is confirmed by the research of Podkówka and Podkówka [28], which recorded a significantly higher starch level for early hybrids (71.3% d.m.) compared to medium-late varieties (70.29% d.m.).

**Table 4:** Starch content in corn grain (g∙kg^-1^) depending on research factors.

Factors studied	Growing season			Average
	2015	2016	2017	
**VARIETIES**				
medium-late—PR38N86	724.41 ^a^	708.71 ^a^	726.89 ^a^	**724,41** ^**A**^
medium-early—P8400	722.23 ^a^	686.84 ^b^	727.14 ^a^	**722.23** ^**B**^
**NITROGEN DOSES**				
Control treatment	731.05 ^a^	699.55 ^a^	725.56 ^a^	**718.72** ^**A**^
80 kg·ha^-1^ N	724.18 ^b^	698.86 ^a^	727.45 ^a^	**716.83** ^**B**^
120 kg·ha^-1^ N	718.55 ^c^	696.15 ^b^	726.63 ^a^	**713.78** ^**C**^
160 kg·ha^-1^ N	719.50 ^c^	696.53 ^b^	728.41 ^a^	**714.81** ^**C**^
**TYPES OF BIOSTIMULATORS USED**				
Control treatment	722.71 ^b^	697.06 ^a^	724.32 ^b^	**714.70** ^**B**^
Biostimulator containing: sodium ortho-nitrophenol, sodium para-nitrophenol, 5-nitroguaiacol sodium	723.00 ^b^	699. 03 ^a^	729.15 ^a^	**717.06** ^**A**^
Biostimulator containing: potassium para-nitrophenolate, potassium ortho-nitrophenolate, potassium 5-nitrovacollate	725.56 ^a^	698,01 ^a^	725.38 ^b^	**716.32** ^**A**^
Biostimulator containing: molybdenum, zinc	722.00 ^b^	696.8 ^a^	729.22 ^a^	**716.07** ^**A**^
** Means for years**	**723.32** ^**B**^	**697.77** ^**C**^	**727.02** ^**A**^	-

* The means for growing seasons, varieties, nitrogen doses, and types of biostimulators used, marked with capital letters A, B, C, differ significantly at p≤0.05. Means for interactions: years and varieties, years and nitrogen doses; years and types of biostimulators used, marked with lowercase letters a, b, c, d differ significantly at p≤0.05.

Own research showed a significant effect of nitrogen doses on starch reducing content (Table 4), which is consistent with the results of Bogucka et al. [11], where increasing nitrogen doses from 0 to 270 N kg∙ha^-1^ resulted in a decrease in the starch content in corn grain. In own research, the lowest starch content was obtained after applying 120 and 160 kg∙ha^-1^ N. Analysis of variance showed a significant interaction between years and nitrogen fertilization. In the first year of the study, the doses of nitrogen applied resulted in a significant reduction in starch content compared to the control object. In this season, after applying 120 and 160 kg∙ha^-1^ N, the starch content in corn grain was the same. A similar tendency was noted in the second year of the study, where statistically insignificant differences were found after applying a dose of 80 kg N∙ha^-1^ on the control object and at 120 and 160 kg kg∙ha^-1^ N. In the last, rather wet year of research, the same value of this feature was recorded in all objects.

Kierzek et al. [33] in their research showed no significant impact positive of the use of Aminoplant and Asahi SL biostimulators on the starch content in corn grain. The growth bioregulators used in the experiment resulted in a reduction in the value of this feature. This is consistent with the results of own research, which proved the influence of the tested factor on the starch content (Table 4). The biostimulators tested in own experiment increased the starch content by an average of 1.19 g∙kg^-1^. It was shown that the differences between the objects on which biostimulators were used were statistically insignificant. Similar results were obtained by Abdel-Lattif et al. [32].

Statistical calculations showed an interaction of years with the types of biostimulators used. In the first season, only the preparation containing potassium para-nitrophenolate, potassium ortho-nitrophenolate, and potassium 5-nitroviacolate increased the starchcontent in corn grain compared to the control. In the second, rather dry year of the study, the value of the tested feature in the objects with biostimulators was the same as in the control object. In the last test period, a biostimulator containing sodium ortho-nitrophenol, sodium para-nitrophenol, sodium 5-nitroguaiacol, molybdenum and zinc increased the starch concentration by an average of 4.8 g∙kg^-1^.

Humidity and thermal conditions determined the starch content. The highest content of the discussed feature was recorded (on average– 727.02 g∙kg^-1^) in the last year of the study, which was characterized by the highest sum of atmospheric precipitation during the growing season, and the lowest–on average 697.77 g∙kg^-1^ in the very dry growing season 2016 (Table 3), which is confirmed by the research of Korniewicz et al. [27] and Niedziółka and Szymanek [35], whose research proved the significant impact of weather conditions on the value of the discussed feature. Moreover, an interaction between years and varieties was demonstrated, which idnciates different responses of varieties to changing climatic conditions in individual growing seasons. The largest statistically significant differences between varieties were found in the second year of the study. The PR38N86 variety contained an average of 21.87 g∙kg^-1^ more protein than P8400.

### Crude fat content in corn grain

Own research showed that the content of crude fat depended on the genetic factor (Table 5). Among the examined varieties, the medium-early variety P8400 had the significantly highest content of the discussed feature–on average 39.55 g∙kg^-1^, while the medium-late variety had, on average, 1.50 g∙kg^-1^ less fat in the grain, which was confirmed by the results of research by Podkówka and Podkówka [28], who obtained the significantly highest value of the discussed feature for medium-early varieties–on average 44.20 g∙kg^-1^. The significantly lower fat level found in medium-late varieties was not confirmed in the study by Korniewicz et al. [27], which found no relationship between the level of this component and the degree of earliness. However, Górny [36] in his research showed significant genetic variability of the fat content in corn grain, which ranged from 3 to 7% of dry matter.

**Table 5:** Crude fat content in corn grain (g∙kg^-1^) depending on research factors.

Factors studied	Growing season			Average
	2015	2016	2017	
**VARIETIES**				
medium-late—PR38N86	36.7 ^b^	38.23 ^b^	39.21 ^a^	**38.05** ^**b**^
medium-early—P8400	38.69 ^a^	40.51 ^a^	39.46 ^a^	**39.55** ^**a**^
**NITROGEN DOSES**				
Control treatment	37.79 ^b^	38.23 ^c^	38.85 ^b^	**38.29** ^**C**^
80 kg·ha^-1^ N	37.80 ^b^	39.51 ^b^	39.13 ^b^	**38.81** ^**B**^
120 kg·ha^-1^ N	38.38 ^a^	39.63 ^b^	39.63 ^a^	**39.21** ^**A**^
160 kg·ha^-1^ N	36.80 ^c^	40.10 ^a^	39.75 ^a^	**38.89** ^**B**^
**TYPES OF BIOSTIMULATORS USED**				
Control treatment	37.25	38.80	38.83	**38.29** ^**B**^
Biostimulator containing: sodium ortho-nitrophenol, sodium para-nitrophenol, 5-nitroguaiacol sodium	38.05	39.86	39.57	**39.16** ^**A**^
Biostimulator containing: potassium para-nitrophenolate, potassium ortho-nitrophenolate, potassium 5-nitrovacollate	37.62	39.41	39.68	**38.90** ^**A**^
Biostimulator containing: molybdenum, zinc	37.86	39.42	39.29	**38.85** ^**A**^
** Means for years**	**37.69** ^**B**^	**39.37** ^**A**^	**39.34** ^**A**^	-

* The means for growing seasons, varieties, nitrogen doses, and types of biostimulators used, marked with capital letters A, B, C, differ significantly at p≤0.05. Means for interactions: years and varieties, years and nitrogen doses; years and types of biostimulators used, marked with lowercase letters a, b, c, d differ significantly at p≤0.05.

What is more, the experiment showed that nitrogen doses significantly increased the crude fat content in corn seeds (Table 5), which was not confirmed by the results of the research by Bogucka et al. [11]. These authors found the highest crude fat content at doses of 30 and 240 N kg∙ha^-1^, while at the remaining nitrogen remaining doses this feature decreased significantly. Also the research by Szulc et al. [37] and Szulc [30], Księżak et al. [4] demonstrated a decrease in crude fat content with an increase in nitrogen dose, but these differences were statistically insignificant.

Own research showed that in the first year of the study, the highest crude fat content was obtained after the application of 120 kg∙ha^-1^ N. In this season, after the application of 80 kg∙ha^-1^ N, the same fat content was obtained as in the control object. In 2016, the highest concentration of crude fat was obtained under the influence of 160 kg∙ha^-1^ N, and the differences in the value of this feature between the nitrogen dose of 80 and 120 kg∙ha^-1^N were statistically insignificant. A similar tendency was noted in the last year of research between doses of 120 and 160 kg∙ha^-1^ N.

Kierzak et al. [33] in their research proved that the Asahi SL and Aminoplant biostimulators did not show a significant effect on the crude fat content, however, slight tendencies to increase the value of this feature by an average of 0.07% compared to the control product in corn grain were observed. Also the study by Michalski [34] confirmed that biostimulators did not significantly affect the content of the trait in question in corn grain, and Abdel-Lattif et al. [32] showed a significant impact of natural stimulants on the value of this feature. Own research showed that the biostimulators used slightly but statistically significantly increased the value of this feature.

Own research confirmed the significant impact of meteorological conditions on the content of crude fat in corn grain (Table 5). The highest crude fat content (39.37 g∙kg^-1^) was obtained in the second driest and warmest year of the study, which is consistent with the reports of Królikowski [38], who believes that the content of chemical compounds in corn grains depends mainly on the prevailing meteorological conditions during the plant’s vegetation. It was found that the varieties responded differently to weather conditions in particular years of the study. The PR38N86 and P8400 cultivars accumulated the least amount of crude fat in the grain in the first year of the study, in PR38N86 the highest value of the discussed feature was recorded in the third year of the study, and the differences between the genotypes in this season were statistically insignificant. The highest crude fat content in the medium-early variety was found in the second year of the study.

## Conclusions

1. Different doses of nitrogen used in the experiment contributed to an increase in the total protein content in the grain of the tested corn varieties compared to the control treatment. The highest significant increase in protein content by an average of 18.96 g∙kg^-1^ was obtained after the application of 160 kg·ha^-1^ N, and after the application of 120 kg·ha^-1^ N the value of this feature increased on average by 17.18 kg·ha^-1^ N.
2. Nitrogen fertilization reduced the starch content and increased the fat content in the tested corn varieties. The lowest starch content was found after the application of 120 kg kg·ha^-1^ N and 160 kg·ha^-1^ N, while the highest fat content was found in grain tested with 120 kg·ha^-1^ N
3. After using a biostimulator containing sodium ortho-nitrophenol, sodium para-nitrophenol, sodium 5-nitroguaiacol, the protein content increased by an average of 6.0%.
4. The biostimulators used increased the starch and fat content in the grain of the tested varieties, and the differences between them were statistically insignificant.
5. The medium-early variety with FAO 240 was characterized by a higher content of total protein (on average by 7.95 g∙kg^-1^) and crude fat (on average by 1.5 g∙kg^-1^) compared to the medium-late variety.
6. All tested varieties were distinguished by the highest concentration of total protein and crude fat in the second, optimal year of the study. The lowest protein content was found in the last year of the study with the highest annual rainfall and the lowest average air temperature, and crude fat content was found in a relatively dry growing season. The opposite tendency was obtained in the case of starch content in the grain of the tested varieties.
